# Supplementary material for: Microbial community composition variation in relation to malaria infections in Junco hyemalis
Source: PLoS One. 2026 Feb 13;21(2):e0340635. doi: 10.1371/journal.pone.0340635 (PMC12904453; doi:10.1371/journal.pone.0340635)
Supplement: Attachment — Submitted filename: Amaya-Mejia 2025.docx [file pone.0340635.s001.docx]

Summary:

This study by Amaya-Mejia and colleagues describes the alteration of gut microbiota in Juncos birds affected by Plasmodium. This paper is describing changes in microbiome associated to the infection, to discuss two main hypothesis about the link between dysbiosis and parasitic infection.

This study is well written, well conducted and very clear. It was a pleasure to read it, and I have no major comments. My only minor suggestions concern the text, and I expect no supplementary experiment.

Although clear, the Results section often lacks an interpretation or a comment after the key results. I made some suggestions hereafter. The introduction presents the challenge of the definition of dysbiosis, but the discussion lacks a follow-up to this introduction.

Please find hereafter my comments.

Abstract:

Line 19 : please rephrase the beginning of the sentence “For beta diversity, while infected birds had different bacteria than non-infected birds” as it is unclear

Line 28: the statement “However, in addition to the observed reduced microbial diversity, dysbiosis requires a significant increase in pathogenic bacteria and reduction of beneficial bacteria.” is unclear

Please use the same appellation for the birds between abstract and keywords, even if it refers to the same bird

Introduction:

Line 41 and later in the text: please either use the new names of Firmicutes (Bacillota) and Actinobacteria (Actinomycetes) or mention them

Line 44-47: could you discuss here the hypothesis of “the parasitic infection is permitted by dysbiosis”? You reject this hypothesis in the discussion, but it seems to me that it is missing from the introduction.

Line 48-50: can you add the notion of functions that are lost with dysbiosis?

Can you add a word about the consequences for Juncos of the parasite infection? Are they strictly carrier/vectors, do they suffer from the infection?

Last paragraph: not sure to follow how the different results would support one or the other hypothesis. This is clearer in the discussion part, notably with the “The hypothesis that gut microbiomes are altered due to Plasmodium infections is further supported by our co-occurrence networks that show significant dissimilarity between infected and non-infected birds, more than is expected by random chance.”, so please rephrase the introduction part to present more clearly how the results could support one or the other hypothesis.

Methods:

Could you provide the sequence of the primers used for the nested PCRs

Results:

The results section often lacks an interpretation or a comment after the key results. I made some suggestions hereafter.

Could you comment the following results “Based on microscopy, parasitemia for these eight infected birds ranged from 0.1 – 0.7% infected RBC.” indicating if the values correspond to what is expected?

Concerning body condition of the birds, could you explicit what is the body condition in terms of health for the bird? It would be clearer to me if you add a comment after the “Body condition, based on residuals of tarsus length and body mass, increased significantly with parasitemia” statement, to explicit the impact on the parasitemia on the bird health. If it’s relevant, you can also add a Figure showing the correlation.

Discussion:

The Discussion lacks a part about the definition of dysbiosis, and the limitation of using 16S sequencing compared to WGS. In the introduction, you discuss the definition of dysbiosis but never address it in the discussion. Moreover, the dysbiosis is a loss of microbial functions more than a decrease of diversity. The discussion is lacking this aspect, and this should be addressed in the limitations section as it is the main limitation of the study.

Line 311: “While Firmicutes have also been reported to be depleted in infected birds, this was not observed in our study.” The Figure 2 seems to indicate a decrease in Firmicutes. There is no comment about this result in the Results section, so could you add something about the non-significant difference that you propose?

In the Limitations section, please add a sentence about the fact that only males were used.

Figures:

Please either use the new names of Firmicutes (Bacillota) and Actinobacteria (Actinomycetes) or mention them

Typos:

Line 37: please remove the colon in the following sentence “In birds and other animals, these functions can include: nutritional uptake, detoxification, immune functions, and potentially serve to outcompete pathogenic microbes” to fit the syntax of the sentence

Line 55: please remove the can “but these can may not capture subtle variations and can be subject to sampling bias”

Line 57: please correct “it may be prudent to account for the relationships observed between bacteria, through co-occurrence networks”

Line 145: please correct “DNA extractions were also used”
